# Online Game Addiction and the Level of Depression Among Adolescents in Manila, Philippines

**DOI:** 10.5195/cajgh.2020.369

**Published:** 2020-03-31

**Authors:** Ryan V. Labana, Jehan L. Hadjisaid, Adrian R. Imperial, Kyeth Elmerson Jumawid, Marc Jayson M. Lupague, Daniel C. Malicdem

**Affiliations:** 1 Department of Biology, College of Science, Polytechnic University of the Philippines, Manila, Philippines; 2 Senior High School, Polytechnic University of the Philippines, Manila, Philippines

**Keywords:** Mental health, Public health, Addiction, Video games, Depression, Neuroscience

## Abstract

**Introduction:**

World Health Organization recognizes online game addiction as a mental health condition. The rise of excessive online gaming is emerging in the Philippines, with 29.9 million gamers recorded in the country. The incidence of depression is also increasing in the country. The current correlational analysis evaluated the association between online game addiction and depression in Filipino adolescents.

**Methods:**

A paper-and-pencil self-administered questionnaire assessing depression and online game addiction was distributed from August to November, 2018. The questionnaire included socio-demographic profiles of the respondents, and the 14-item Video Game Addiction Test (VAT) (Cronbach's α=0.91) and the Patient Health Questionnaire-9 (Cronbach's α=0.88) to determine levels of online game addiction and depression, respectively. Multiple regression analyses were used to test the association between depression and online game addiction.

**Results:**

Three hundred adolescents (59% males, 41% females) participated in the study. Fifty-three out of 300 respondents (12.0% males, 5.7% females) had high level of online game addiction as reflected in their high VAT scores. In this study, 37 respondents (6.7% males, 5.7% females) had moderately severe depression and 6 (2.0%) females had severe depression. Online game addiction was positively correlated with depression in this study (*r*=0.31; *p*<0.001). When multiple regression analysis was computed, depression was found to be a predictor of online game addiction (*Coefficient*=0.0121; 95% CI-8.1924 - 0.0242; *p*=0.05).

**Conclusions:**

Depression, as associated with online game addiction, is a serious threat that needs to be addressed. High level of online game addiction, as positively correlated to the rate of depression among adolescents in Manila, could potentially be attributed to the booming internet industry and lack of suffiicent mental health interventions in the country. Recommended interventions include strengthening depression management among adolescents and improving mental health services for this vulnerable population groups in schools and within the communities.

Based on the report of the European Mobile Game Market in 2016, there were more than 2.5 billion video gamers across the globe.^1^ Several studies have found that the majority of these players were adolescents aged 12-17 years,^2^-^5^ with more usage among males than females.^6^ In 2017, newzoo.com reported that the active gamers in the Philippines were 52% males and 48% females.^7^ In the US, 60% of the video gamers were males and 40% are females.^6^ Studies have shown that there are similarities between males and females in regard to choice of games, behavior toward video gaming, and motives for engaging in this activity.^8^ Some of the reported reasons to engage in video games include having fun and for recreation,^9^-^10^ to de-stress,^11^^–^^12^ and to avoid real life issues.^13^^–^^14^ The prevalence of video gaming addiction varies from region to region based on the socio-cultural context and the criteria used for the assessment.^15^ However, it is well established that video gaming is addictive,^16^^–^^18^ and there is clinical evidence for the symptoms of biopsychosocial problems among video game addicts.^19^ It is a serious threat to the mental and psychosocial aspects of an individual, as it lead to stress, loss of control, aggression, anxiety, and mood modification.^20^^–^^21^

In the Philippines, online gaming is an emerging industry. The country ranks 29^th^ in game revenues across the globe. In 2017, there were more than 29.9 million gamers recorded in the country. Most of the gamers were 21–35 years of age, followed by the adolescents 10–20 years of age.^7^ Adolescents accounted for 30.5% of the total population in the country.^22^ In general, this age group is already facing mental health issues, such as anxiety, mood disorders, and depression. This concern gets more alarming as rates of suicide among high school and college students are growing worldwide.^23^

World Health Organization lists video game addiction as a mental health problem.^24^ Psychiatric research reported evidence on the links between depression and video game addiction. Among the findings are MRI scans of video game addicts showing disruption of some brain parts and overriding of the 'emotional' part with the 'executive' part.^25^ A study in China has also reported that gamers are at increased risk of being depressed in comparison to those who did not play video games.^26^ In the field of neuroscience, depression caused by online game addiction is explained as a reduction of synaptic activities due to permanent changes in the dopaminergic pathways. This means that long exposure to online gaming causes changes in a person's sense of natural rewards, often making activities less pleasurable. This neuroadaptation is also associated with chronic depression.^27^

There is a paucity of studies on video game addiction in the Philippines, making its implications not well understood. There are reports of the impact of video game addiction on the academic performance of the gamers,^28^^–^^30^ but no study has been found associating video game addiction and depression in the Philippine setting. Based on the 2004 report from the Department of Health in the Philippines, over 4.5 million cases of depression were reported in the country. Recently, World Health Organization reported that 11.6% of the 8,761 surveyed young Filipinos considered committing suicide; 16.8% of them (of 8,761) had attempted it.^31^ This phenomenon is said to be instigated by several factors, including the individual's exposures to technology. Video game addiction and depression are two emerging public health issues among adolescents in the Philippines.^31^^–^^32^ This small-scale study aims to understand the association between these two factors and produce baseline information that can be used in formulating evidence-based public health policies in the country.

## Methods

### Research site and participants

This study was conducted in the months of August-November 2018 in the city of Manila, the capital of the Philippines. Manila is situated on the eastern shores of Manila Bay, on the western edge of Luzon (14∘35’45”N 120∘58’38”E). It is one of the most urbanized areas and the center of technological innovation in the country. It has a population of 1.78 million, based on 2016 census.^33^ Manila covers 896 barangays (villages), which are grouped into six districts. Based on the 2010 census, the total population of Filipino adolescents, regardless of sex, was 166,391.^34^ This population estimate was used for computing the sample size needed for this study. Sample size calculation was estimated using the online calculator from OpenEpi.^35^ The completion rate of the questionnaires was 78.13%, for a total of 300 consenting respondents who were all online video gamers. They were selected if they were residents of Manila City and reported playing video games on the regular basis.

**Figure 1.**
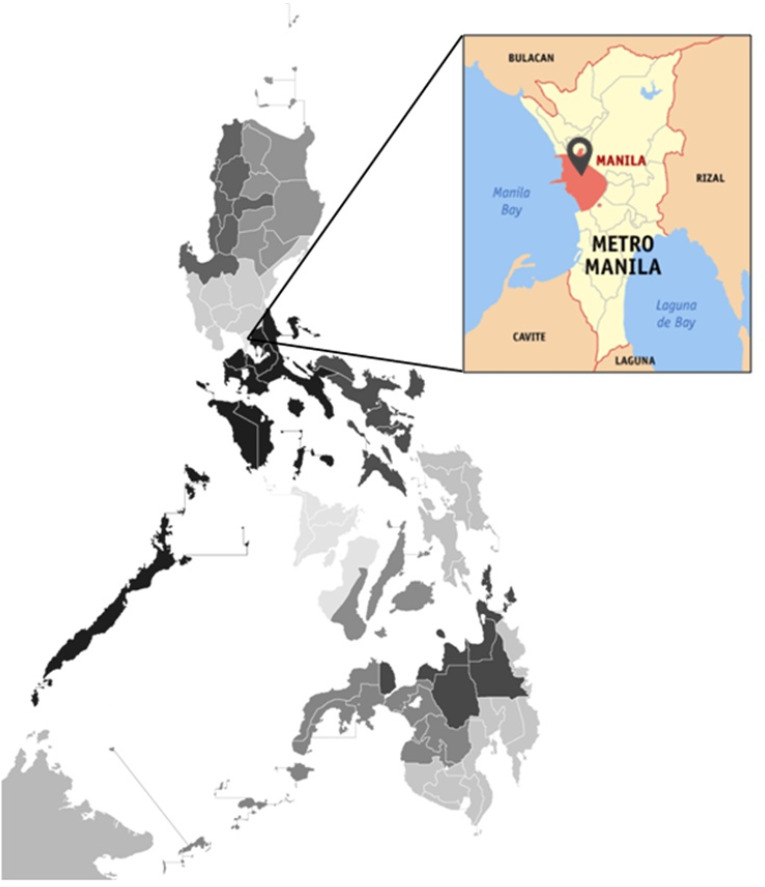
Map of Manila from the National Capital Region of the Philippines

### Instruments

The study used a paper-and-pencil self-administered questionnaire. To determine the level of online game addiction of the respondents, the study used the Video Game Addiction Test (VAT) developed by van Rooij *et al*.^36^ from the 14-item version of the Compulsive Internet Use Scale (CIUS).^37^ VAT was utilized in several studies among adolescents in the past, and it has demonstrated excellent reliability and validity. The scale outcomes were found to be comparable across gender, ethnicity, and learning year, making it a helpful tool in studying video game addiction among various subgroups.^36^ The survey contains questions in five categories: loss of control, conflict, salience, mood modification, and withdrawal symptoms. Each question was measured on a 5-point scale: 0–never to 4–very often. The results were then used as an indicator of the level of addiction. This study adapted the calculations conducted by van Rooij *et al.*^38^ wherein the average scale scores of all the respondents were arranged from 0-4 and then were divided into two groups. The first group had an average of 0-2 or 'never' to 'sometimes', while the second group had an average of 3-4 or 'often' to 'very often'. The latter group was considered to have the highest level of problematic gaming or, in this study, with online game addiction.^38^ The internal reliability of the VAT in this study was excellent at Cronbach's α of 0.91.

The level of depression of the respondents was determined by using the Patient Health Questionnaire-9 (PHQ-9).^39^ It is a 9-item depression module taken from the full PHQ. The questionnaire allows the respondents to rate their health status in the past six weeks. There are 9 diagnostic questions in which the respondents rated 0 for 'not at all', 1 for 'several days', 2 for 'more than half the days', and 3 for 'nearly every day'. The total of the PHQ-9 scores was used to measure severity of depression. Since there are 9 items in the questionnaire and each question can be rated from 0-3, the PHQ-9 scores can range from 0-27. The score was interpreted as ‘no depression’ (0-4 points), ‘mild depression’ (5-9 points), ‘moderate depression’ (10-14 points), ‘moderately severe depression’ (15-19 points), and ‘severe depression’ (20-27 points).^39^ In this study, the internal reliability of PHQ-9 had a Cronbach's α of 0.88.

### Data gathering procedure

The study randomly surveyed gamers in various parts of Manila. Since there are no reliable records of the gamers in the area available for research, various sampling techniques were utilized. A convenience sampling was done by visiting internet cafes in the city and requesting the gamers to answer the questionnaire during their time-out (from the game). A verbal consent was provided by each respondent after hearing a brief explanation of the research objectives and the necessary instructions. While answering the questionnaire, the respondents were assisted by the investigator for any clarifications and questions. The questionnaire was completed by the respondents in approximately 2.5 minutes. Other procedures included snowball sampling, accidental, and voluntary response sampling after the distribution of invitation to respond among internet cafes, gamers’ social media groups/sites, and online gamers’ organizations. The study was approved by the ethical board of the Polytechnic University of the Philippines.

### Statistical analysis

All the responses from the questionnaires were inputted into MS Excel and into SPSS version 23.0 (IBM Corp., Armonk, NY, USA). Descriptive statistics of responses were computed and included the frequencies (*f*), percentages (%), averages x and standard deviations (SD). The association between online game addiction and depression was analyzed using Pearson's correlation and was further analyzed using a multiple regression analysis. The study hypothesized that there is no significant correlation between online game addiction and level of depression among adolescents in the City of Manila, Philippines. All statistical results were considered significant at the *p* value <0.05.

## Results

### Profile of the respondents

**Figure 2.**
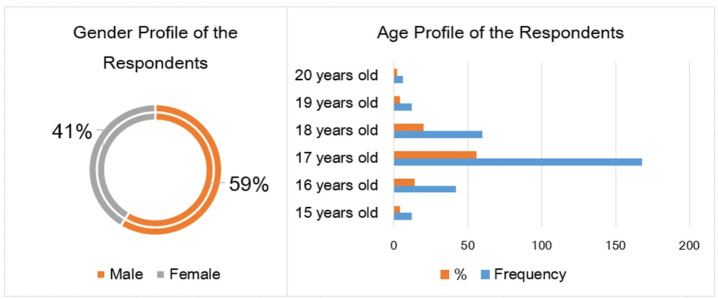
Profiles of the respondents based on gender and age

A total of 300 consenting adolescents participated in the study. There were more males (*n*=176; 59%) than females (*n*=124; 41%) who participated in the study. Most of the respondents were adolescents (aged less than 19 years), except for the six respondents who were already 20 years old during the data gathering. The mean age of the participants was 17 years old (SD=0.90). Figure 2 presents the profiles of the respondents based on their gender and age characteristics. The VAT analysis shows that there were more males (12.0%) who were addicted to online games than females (5.7%). Meanwhile, 15-, 17-, and 18-year old respondents had the highest VAT scores among the six age groups.

### Level of online game addiction

The 14-item VAT was ranked from the highest to the lowest mean score to understand the common conditions experienced by the respondents. The item with the highest mean was No. 13: *Do you game because you are feeling down?* (x=2.1, SD=1.40). This question had the third greatest number of “4-very often” ratings (*N*=46/300). It was followed by the item No. 3: *Do others (e.g., parents or friends) say you should spend less time on games?* (x=2.06, SD=1.41).. The third item with the highest mean score was item No. 7: *Do you look forward to the next time you can game?* (x=2.0, SD=1.27). The item with the highest number of “4-very often” rating was item No. 14: *Do you game to forget about problem?* (*N*=67/300). Items 12 and 2 also had high mean scores: *Do you neglect to do your homework because you prefer to game?* (Item 12; x=1.98, SD=1.34); and *Do you continue to use the games despite your intention to stop*? (Item 2; x=1.84, SD=1.20).

**Table 1.** Levels of online game addiction based on gender and age

Profiles	Overall Profile		Respondents with high VAT scores^a^		Respondents with high VAT scores^b^	
*N*	%	*N*	%	*N*	%
**Gender**						
Male	176	58.7	36	12.0	36	20.5
Female	124	41.3	17	5.7	17	13.7
**Age**						
15 years old	12	4.0	3	1.0	3	25.0
16 years old	42	14.0	3	1.0	3	7.1
17 years old	168	56.0	34	11.3	34	22.0
18 years old	60	20.0	12	4.0	12	20.0
19 years old	12	4.0	1	0.3	1	8.3
20 years old	6	2.0	0	0.0	0	0.0

a Percentage was computed against the overall number of participants (*N*=300)

b Percentage was computed against *N* of each profile of the respondents

### Level of depression

The PHQ-9 was used to quantify the symptoms of depression of the respondents and identify its severity. The majority of the respondents demonstrated no depression (47%), followed by having mild depression (22%), and moderate depression (17%). Of note, the current study revealed 12% of the respondents had moderately severe depression and 2% had severe depression. We found that higher PHQ-9 scores were associated with decreased functional status. The most common symptoms reported by the respondents based on the mean scores of each item in PHQ-9 include *…feeling tired or having little energy* (x=1.89, SD=1.30), * …poor appetite or overeating* (x=1.87, SD=1.37), … *feeling down, depressed or hopeless* (x=1.81, SD=1.18), *…trouble falling or staying asleep, or sleeping too much* (x=1.78, SD=1.33), and *…trouble concentrating on things, such as reading newspaper or watching television* (x=1.75, SD=1.40). Interestingly, the six respondents who were identified to have “severe” depression were all females, and four of them had high VAT scores.

**Table 2.** Level of depression of the respondents based on the PHQ-9 scores

Profiles	No Depression	Mild	Moderate	Moderately Severe	Severe
	*N* (%)	*N* (%)	*N* (%)	*N* (%)	*N* (%)
**Gender**					
**Male**	87 (29.0)	38 (12.7)	31 (10.3)	20 (6.7)	0 (0.0)
Female	54 (18.0)	28 (9.3)	19 (6.3)	17 (5.7)	6 (2.0)
Age					
15 years	5 (1.7)	1 (0.3)	2 (0.7)	2 (0.7)	0 (0.0)
16 years	24 (8.0)	9 (3.0)	3 (1.0)	7 (2.3)	0 (0.0)
17 years	77 (25.7)	34 (11.3)	31 (10.3)	22 (7.3)	5 (1.7)
18 years	26 (8.7)	17 (5.7)	10 (3.3)	5 (1.7)	1 (0.3)
19 years	5 (1.7)	4 (1.3)	3 (1.0)	1 (0.3)	0 (0.0)
20 years	4 (1.3)	1 (0.3)	1 (0.3)	0 (0.0)	0 (0.0)

### Association between online game addiction and depression

The association between online game addiction based on the VAT scores and the level of depression among the respondents was evaluated through Pearson's correlation analysis. Results (Table 3) show that the level of online game addiction was positively correlated with the level of depression (*r*=0.31, *p*<0.001) but was not significantly correlated with age or gender (*r*=-0.80, *p*<0.171 and *r*= 0.10, *p*<0.097, respectively).

**Table 3.** Pearson's correlation coefficient among gender, age, online game addiction, and depression of the adolescents in Manila

Variables	Gender	Age	Online game addiction	Depression
**Gender**	1	−.080	.100	.070
		.171	.097	.212
**Age**	−.080	1	−.080	−.020
	.171		.171	.739
**Online game**	.100	−.080	1	.310
**addiction**	.097	.171		.000^*^
**Depression**	.070	−.020	0.310	1
	.212	.739	.000^*^		

* significant at *p*≤.0.001 in correlation matrix

A multiple linear regression was calculated to predict online game addiction based on gender and depression. This regression analysis was performed with all participants and with the subset of participants with high VAT scores, which indicated online game addiction. A significant regression equation was found (F(2.50)= 2.247, 0.10), with an *R*^2^ of 0.082. Table 4 shows that depression was a significant predictor of online game addiction.

**Table 4.** Multiple regression analysis for prediction of online game addiction based on age and level of depression

Variable	Regression coefficient	95% CI	*p* value
**Adolescents playing online games (N=300)**			
Age	–0.0224	–0.1298 – 0.0850	0.68
Depression	0.0418	0.0271 – 0.0565	0.47
**Adolescents with addiction playing online games (n=54)**			
Age	–0.0443	–0.1305 – 0.0417	0.30
Depression	0.0121	–8.1924 – 0.0242	0.05^*^

* significant at *p*<0.05

## Discussion

The correlation between online game addiction and the levels of depression in this study was weak but statistically significant. This positive correlation was previously reported in other research studies across the globe.^40^^–^^41^ In a study conducted by Rikkers *et al.*^40^ among children and adolescents (11-17 years old) in Australia, electronic gaming was positively associated with emotional and behavioral problems including depression. Longer gaming hours were also associated with severe depressive symptoms, somatic symptoms, and pain symptoms among young people in Taiwan.^41^ Online game addiction was associated by Zamani *et al.*^42^ not only with depression but also with sleep disorder, physical complaints, and social dysfunctions of students in Iran. In a study conducted by Dong *et al.,*^43^ depression came out as one of the outcomes of the internet addiction disorder.

In the current study, most of the respondents looked forward to the next time they would game, with the most common reason of engaging in games reported to be easing the moments of feeling down. Another reason of the respondents’ addiction to online games was that they want to forget about problems. It is considered as one of the core symptoms of addiction as described by Brown.^44^ The second most common experience of the respondents was the 'inability to voluntarily reduce the time spent on online games', which is another core symptom of addiction.^45^ Most of the respondents admitted that they were getting advice from their parents or friends to spend less time on games, but they could not control it, despite their intention to stop. In fact, gaming negatively affected homework completion among many study participants. This effect was previously studied among high school students in Los Baños, Philippines, where the video gamers had 39% probability to fail in school. In this previously published study, 6 out of 10 video gamers spent their daily allowances on computer games, giving them access to continuously spend their time playing.^29^ The addiction of the adolescents in Manila could have been influenced by the ubiquitous nature of internet in the city. Internet cafes are very accessible in the country, and they are thriving in almost all corners of the city. In addition, the rent for internet and online games in Metro Manila costs 10 to 20 pesos per hour only (US $0.19 to US $0.38 per hour), making playing video games affordable. Some internet hubs are even offering discounts and promotions for longer stays of 10-12 straight hours of playing online games.

Based on the most cited symptoms of the respondents in this study, it could be implied that adolescents cope with their emotional distress by playing online games. This means that the high occurrence of online game addiction goes along with the high occurrence of depression among the same group. In regard to depression, most respondents in this study were feeling tired, having poor appetite, feeling hopeless, having trouble falling asleep, or having trouble concentrating on things that require enough attention, like reading books. These symptoms were also reported by Schmit *et al.*^45^ as related to online game addiction, where the people who spent longer hours playing online games got higher scores for loneliness and isolation. This study did not capture the number of hours spent by the respondents in online games, which could be incorporated in the next study for further analysis.

Depression, as associated to online game addiction, may lead to anxiety, compulsion, and suicide ideations.^46^ This is a serious threat to the population health that needs to be addressed. Interventions may include strengthening depression management among adolescents, either in school or in the community. There are several ways to manage depression. The schools and the community should reinforce sports by making it more challenging, engaging, and motivating. In the Philippines, numerous factors make receiving mental health care a challenge. There is only one psychiatrist for every 250,000 mentally ill patients, budget dedicated to mental health interventions is limited,^47^ a guidance and counseling system has not yet matured,^48^ and there was even a report that online counseling was preferred by the students than its face-to-face counterpart.^49^ The poor availability of the mental health interventions in the country may lead to upsurge of depression cases among adolescents. Meanwhile, the booming online game industry in the country leads to the increased numbers of addicted adolescents to online game addiction. Policy makers, the government, and its stakeholders should start addressing these issues before it becomes an even bigger health concern, especially in the face of ongoing COVID-19 pandemic.

The Philippine government should also assess their existing intervention programs in mental health issues. In 2016, “Hopeline” was launched in the Philippines. It was a national hotline for mental health assistance for the prevention of depression and suicide cases in the country. The hotline is equipped with a professional team of counselors as responders.^50^ No study has been found to assess the effectiveness of this intervention for depression. National trainings and workshop programs have been implemented in other countries to empower the people in dealing with the stigma of depression which includes mental health literacy campaign, peer services, and advocacies.^51^ This is an essential step to correct various misconceptions on depression, especially among adolescents.

This study was cross-sectional and cannot determine causality. This is the first report on the association between online game addiction and the levels of depression among adolescents in the city of Manila, Philippines. Despite the small sample and the limited scope of the research, the current study has shown interesting preliminary results that could be instrumental in the conduct of a bigger scale study in the country. To facilitate participation of the larger number of respondents, the future investigators are suggested to coordinate with various high schools in Metro Manila and use these schools as a sampling frame for a robust sampling technique. In this study, the level of online game addiction has no statistically significant association with age and gender. The association between age and online game addiction could have been improved by including older age groups in this study. Data from a group of young adults (college students), who are also exposed to online gaming, could be compared to these data for further analysis. Gender is commonly associated with the level of online game addiction in many studies, but it is not statistically significant in this present study. The sample size in this study was only 300 and may not have been representative enough of a general population. Also, our sample size was not large enough to capture distinctions between males and females. This could also be addressed by a wider scale of surveys in the future research.

## References

[1] ePC. 2019 Video Game Industry Statistics, Trends & Data [Accessed 2020 June 15]. Available from https://www.wepc.com/news/video-game-statistics/.

[2] Griffiths MD, Davies MN, & Chappell D. Online computer gaming: a comparison of adolescent and adult gamers. J Adolesc 2004; 27: 87–96. doi: 10.1016/j.adolescence.2003.10.007. 15013262

[3] Barnett J & Coulson M. Virtually real: A psychological perspective on massively multiplayer online games. Review of General Psychology 2010; 14: 167–179. doi: 10.1037/a0019442.

[4] Elson M & Ferguson CJ. Gun violence and media effects: Challenges for science and public policy. The British Journal of Psychiatry 2013; 203: 322–324. doi: 10.1192/bjp.bp.113.128652. 24187065

[5] Ferguson CJ, Coulson M, & Barnett J. A meta-analysis of pathological gaming prevalence and comorbidity with mental health, academic and social problems. Journal of Psychiatric Research 2011; 45: 1573–1578. doi: 10.1016/j.jpsychires.2011.09.005. 21925683

[6] Statista. Distribution of computer and video gamers in the United States from 2006 to 2019, by gender. [Accessed 2020 June 15]. Available from https://www.statista.com/statistics/232383/gender-split-of-us-computer-and-video-gamers/

[7] Newzoo. The Filipino Gamer, 2017 [Accessed 2020 June 15]. Available from https://newzoo.com/insights/infographics/the-filipino-gamer/.

[8] Veltri NF, Krasnova H, Baumann A, & Kalayamthanam N. Gender differences in online gaming: A literature review. Proceedings of the 20^th^ Americas Conference on Information Systems, Savannah, 2014 [Accessed 2020 June 15]. Available from http://citeseerx.ist.psu.edu/viewdoc/download?doi=10.1.1.667.4530&rep=rep1&type=pdf.

[9] Griffiths MD & Hunt N. Computer game playing in adolescence: Prevalence and demographic indicators. Journal of Community & Applied Social Psychology 1995; 5: 189–193. doi: 10.1002/casp.2450050307

[10] Kuss DJ & Griffiths MD. Internet gaming addiction: A systematic review of empirical research. International Journal of Mental Health and Addiction 2012; 10: 278–296. doi: 10.1007/s11469-011-9318-5

[11] Grusser SM, Thalemann R, Albrecht U, & Thalemann CN. Excessive computer usage in adolescents-a psychometric evaluation. Wiener KlinischeWochenschrift 2005; 117: 188–195. doi: 10.1007/s00508-005-0339-6. 15875758

[12] Wood RTA & Griffiths MD. A qualitative investigation of problem gambling as an escape based coping strategy. Psychology and Psychotherapy: Theory, Research and Practice 2007; 80: 107–125. doi: 10.1348/147608306X107881. 17346384

[13] Wan, CS & Chiou WB. Why are adolescents addicted to online gaming? An interview study in Taiwan. Cyber Psychology& Behavior 2006; 9: 762–766. doi: 10.1089/cpb.2006.9.762. 17201603

[14] Wood RTA, Griffiths MD, & Parke A. Experiences of time loss among videogame players: An empirical study. CyberPsychology& Behavior 2007; 10: 38–44. doi: 10.1089/cpb.2006.9994. 17305447

[15] Fu KW, Chan WSC, Wong PWC, & Yip PSF. Internet addiction: Prevalence, discriminant validity and correlates among adolescents in Hong Kong. Br. J. Psychiatry 2010; 196: 486–492. doi: 10.1192/bjp.bp.109.075002. 20513862

[16] Griffiths M. Does internet and computer “addiction” exist? Some case study evidence. Cyber Psychology & Behavior 2004;3(2): 211–218. doi: 10.1089/109493100316067.

[17] Griffiths MD, Kuss DJ, & King DL. Video game addiction: Past, present, and future. *Current Psychiatry Review*, 2012; 8:0000–000. doi: 10.2174/157340012803520414.

[18] Griffiths MD. Online games, addiction, and overuse of. In The International Encyclopdia of Digital Communication and Society. 1st ed. John Wiley & Sons, Inc.; 2015. doi: 10.1002/9781118290743.wbiedcs044.

[19] Beutel ME, Hoch C, Woelfing K, Mueller KW. Clinical characteristics of computer game and Internet addiction in persons seeking treatment in an outpatient clinic for computer game addiction. Z. Psychosom. Med. Psychother. 2011, 57, 77–90. doi: 10.13109/zptm.2011.57.1.77. 21432840

[20] Kuss DJ & Griffiths MD. Internet and gaming addiction: A systematic literature review of neuroimaging studies. Brain Sci 2012; 2: 347–374. doi: 10.3390/brainsci2030347. 24961198 PMC4061797

[21] Anderson CA, Funk JB, & Griffiths MD. Contemporary issues in adolescent video game playing: brief overview and introduction to the special issue. J Adolesc 2004; 27(1), 1–3. doi: 10.1016/j.adolescence.2003.10.001.

[22] UNICEF. At a glance: Philippines [Accessed 2020 June 15]. Available from https://www.unicef.org/infobycountry/philippines_statistics.html#123.

[23] Lee YJ, Cho SJ, Cho IH, & Kim SJ. Insufficient sleep and suicidality in adolescents. Sleep 2012; (4):455–60. doi: 10.5665/sleep.1722. 22467982 PMC3296786

[24] Keaten J & Cheng M. Cumpolsive video-game playing could be mental health problem [Accessed 2020 June 15]. Available from https://medicalxpress.com/news/2018-06-compulsive-video-game-mental-health-problem.html.

[25] Hymas C & Dodds L. Addictive video games may change children's brains in the same way as drugs and alcohol, study reveals [Accessed 2020 June 15] Available from https://www.telegraph.co.uk/news/2018/06/12/addictive-video-games-may-change-childrens-brains-way-drugs/.

[26] Peng W & Liu M. Online gaming dependency: A preliminary study in China. CyberpsycholBehav Soc Netw 2010; 13 (3). doi: 10.1089/cyber.2009.0082. 20557254

[27] Craven R. Targeting neural correlates of addiction. Nat Rev Neurosci 2006;7:1. doi: 10.1038/nrn1840.

[28] Verecio R. Online gaming addiction among BSIT students of Leyte Normal University Philippines its implication towards academic performance. INDJSRT 2018; 11(47): 1–4. DOI: 10.17485/ijst/2018/v11i47/137972.

[29] Cortes MDS, Alcalde JV, & Camacho JV. Effects of computer gaming on High School students' performance in Los Baños, Laguna, Philippines. 国 際公共政策研究 (International Public Policy Research). 2012; 16(2):75–88. [Accessed December 2020]. Available from: https://ir.library.osaka.ac.jp/repo/ouka/all/24497/osipp_030_075.pdf.

[30] Lumbay C, Larisma CCM, Centillas Jr. CL. Computer gamers'academic performance in a Technological State College in Leyte, Philippines. Journal of Social Sciences 2017; 6(2):41–49. doi: 10.25255/jss.2017.6.2S.41.49.

[31] Rappler. Mental illness, Suicide Cases Rising Among Youth [Accessed 2020 June 15]. Available from https://www.rappler.com/newsbreak/in-depth/211671-suicide-cases-mental-health-illness-youth-rising-philippines.

[32] Rappler. How does the PH fare in mental health care? [Accessed 2020 June 15]. Available from https://www.rappler.com/newsbreak/iq/184754-philippines-mental-health-care.

[33] World Population Review. Manila Population 2019 [Accessed 2020 June 15]. Available from http://worldpopulationreview.com/world-cities/manila-population/.

[34] Philippine Statistics Authority. Population of the City of Manila Climbed to 1.7 Million (Results from the 2010 Census of Population and Housing) [Accessed 2020 June 15]. Available from https://psa.gov.ph/content/population-city-manila-climbed-17-million-results-2010-census-population-and-housing.

[35] OpenEpi. Open Source Epidemiologic Statistics for Public Health. [Accessed 2020 June 15]. Available from http://openepi.com/SampleSize/SSPropor.htm.

[36] van Rooij AJ, Schoenmakers TM, van den Eijnden RJ, Vermulst AA, & van de Mheen D. Video game addiction test: validity and psychometric characteristics. *Cyber psychol Behav Soc Netw* 2012; 15(9):507–11. doi: 10.1089/cyber.2012.0007. 22900926

[37] Meerkerk GJ, Van Den Eijnden RJ, Vermulst AA, & Garretsen HF. The Compulsive Internet Use Scale (CIUS): some psychometric properties. *Cyber psychol Behav* 2009; 12(1):1–6. doi: 10.1089/cpb.2008.0181. 19072079

[38] van Rooij AJ, Kuss DJ, Griffiths MD, Shorter GW, Shoenmakers TM, van de Mheen D. The (cooccurrence of problematic video gaming, substance use, and psychosocial problems in adolescents. Journal of Behavioral Addictions 2014; 3(3):157–165. doi: 10.1556/JBA.3.2014.013. 25317339 PMC4189309

[39] Kroenke K, Spitzer RL, Williams JB. The PHQ-9: validity of a brief depression severity measure. J Gen Intern Med 2001; 16(9):606–13. PMCID: PMC1495268. 11556941 10.1046/j.1525-1497.2001.016009606.x PMC1495268

[40] Rikkers W, Lawrence D, Hafekost J, Zubrick SR. Internet use and electronic gaming by children and adolescents with emotional and behavioral problems in Australia –results from the second child and adolescent survey of mental health and wellbeing. BMC Public Health 2016; 16:399. doi: 10.1186/s12889-016-3058-1. 27178325 PMC4866411

[41] Weigh H-T, Chen M-H, Huang P-C, Bai Y-M. The association between online gaming, social phobia, and depression: an internet survey. BMC Psychiatry 2012; 12:92 doi: 10.1186/1471-244X-12-92. 22839747 PMC3545926

[42] Zamani E, Chashmi M, & Hedayati N. Effect of addiction to computer games on physical and mental health of female and male students of guidance school in City of Isfahan. Addict Health 2009.; 1(2): 98–104. PMCID: PMC3905489. 24494091 PMC3905489

[43] Dong G, Lu Q, Zhou H, Zhao X. Precursor or sequela: pathological disorders in people with internet addiction disorder. PLoS One 2011; 6(2):e14703. doi: 10.1371/journal.pone.0014703. 21358822 PMC3040174

[44] Brown I. A theoretical model of the behavioral addictions—Applied to offending. In. Hodge JE, McMurran M, Hollins CR. Eds. Chichester, UK: John Wiley; 1997, p. 13–65.

[45] Schmit S, Chauchard E, Chabrol H, & Sejourne N. Evaluation of the characteristics of addiction to online video games among adolescents and young adults. Encephale 2011; 37 (3): 217–223. doi: 10.1016/j.encep.2010.06.006. Epub 2010 Aug 17. 21703437

[46] Wenzel T, Rushiti F, Aghani F, Diaconu G, Maxhuni B, & Zitterl W. Suicidal ideation, post-traumatic stress and suicide statistics in Kosovo. An analysis five years after the war. Suicidal ideation in Kosovo. Torture 2009; 19(3):238–47. PMID: 20065542. 20065542

[47] Newman C. Minding the gap in Philippines' mental health. [Accessed 2020 June 15] Available from https://www.bworldonline.com/minding-gap-philippines-mental-health/.

[48] Lagon HM. Guidance and counseling in the Philippines: A journey to maturity. [Accessed 2020 June 26]. Available from https://archive.dailyguardian.com.ph/guidance-and-counseling-in-the-philippines-a-journey-to-maturity/.

[49] Teh LA, Acosta AC, Hechanova MRM, Alianan AS. Attitudes of Psychology graduate students toward face-to-face and online counseling. Philippine Journal of Psychology 2014; 47 (2): 65–97.

[50] Rappler. National hotline for mental health assistance now open [Accessed 2020 June 15]. Available from https://www.rappler.com/nation/146077-dohhotline-mental-health-assistance-open-suicide-prevention.

[51] National Academies of Sciences, Engineering, and Medicine. (2016). Ending Discrimination Against People with Mental and Substance Use Disorders: The Evidence for Stigma Change. Washington, DC: The National Academies Press. doi: 10.17226/23442. 27631043

